# Prognostic value of hematological parameters in patients with paraquat poisoning

**DOI:** 10.1038/srep36235

**Published:** 2016-11-08

**Authors:** Deng-Chuan Zhou, Hong Zhang, Zhi-Ming Luo, Qi-Xing Zhu, Cheng-Fan Zhou

**Affiliations:** 1 Department of Emergency Medicine, the First Affiliated Hospital, Anhui Medical University, Hefei, Anhui 230022, China; 2 Department of Occupational Health and Environmental Health, School of Public Health, Anhui Medical University, Hefei, Anhui 230032, China; 3 Institute of Dermatology, the First Affiliated Hospital, Anhui Medical University, Hefei, Anhui 230032, China

## Abstract

Paraquat (PQ) is a non-selective contact herbicide, and acute PQ poisoning has a high mortality. The aim of the present study is to evaluate the prognostic value of hematological parameters in patients with acute PQ poisoning. We retrospectively reviewed the records of patients with acute PQ poisoning from January 2010 to December 2015 at the First Affiliated Hospital, Anhui Medical University (Hefei, China). A total of 202 patients were included in the study, and the 30-day mortality was 51.98%. Leukocyte, neutrophil counts and neutrophil-lymphocyte ratio (NLR) were significantly higher in non-survivors than in survivors. In the receiver operating characteristic (ROC) curve analysis, the NLR had an area of 0.916(95%CI, 0.877–0.954) and the optimal cut-off value was 10.57 (sensitivity, 86.70%; specificity, 83.51%; Youden’s index, 0.702). The leukocyte counts had an area of 0.849(95%CI, 0.796–0.902) and the optimal cut-off value was 13.15 × 10^3^/mm^3^ (sensitivity, 77.10%; specificity, 83.50%; Youden’s index, 0.606). The neutrophil counts had an area of 0.878(95%CI, 0.830–0.925) and the optimal cut-off value was 10.10 × 10^3^/mm^3^ (sensitivity, 83.80%; specificity, 79.38%; Youden’s index, 0.632). NLR, leukocyte and neutrophil counts are associated with the 30-day mortality, which may be useful and simple parameters in predicting the prognosis of PQ poisoning.

Paraquat (PQ) is a non-selective contact herbicide, which has been widely used for its high efficiency and low residues in crops^1^. In many developing countries PQ is easily available and inexpensive, which make poisoning prevention difficult^2^. Intentional or accidental ingestion is the major reason associated with PQ poisoning, which many fatalities have been reported each year^3^. Following the ingestion of large amounts of concentrated formulation, PQ has been shown to cause significant damage to multiple organs, including the lung, liver, kidney and myocardium, and the rapid development of multi-organ failure is often lead to fatality^4^. Moreover, even with the ingestion of smaller amounts, PQ is actively taken up through the highly developed polyamine uptake system, which ultimately leads to pulmonary fibrosis and respiratory failure^5^.

Although many investigators have attempted to found efficacious treatments for the management of acute PQ poisoning, but until recently, the clinical therapeutics have been disappointing and the mortality rate still remains very high. Therefore, a reliable predictor of prognosis may be helpful to guide treatment and future clinical research on antidotes and other therapies. At present, several prognostic indicators have been reported to able to evaluate the prognosis of acute PQ poisoning, which including plasma PQ concentration^6^, arterial lactate level and lactate metabolic clearance rate^7^, the Acute Physiology and Chronic Health Evaluation (APACHE) II score^8^, modified Simplified Acute Physiology Score II (MSAPS II)^9^, the Sequential Organ Failure Assessment (SOFA) score^10^, and the Severity Index of Paraquat Poisoning (SIPP)^11^. However, a major constraint of these methods is often unobtainable or unreliable in a number of severely poisoned patients. Therefore, alternative prognostic indicators for acute PQ poisoning are still required for clinical practice.

Although the exact mechanism of PQ toxicity has not been clearly elucidated, it has been extensively indicated that PQ-induced toxicity is due to a sustained redox-cycling and the subsequent generation of reactive oxygen species (ROS), which resulting general inflammatory due to oxidative stress^12^. When the complete blood count (CBC) is evaluated during the acute inflammatory response due to oxidative stress, an increase in leukocytes and neutrophil counts, whereas a decline in lymphocyte counts, were observed^13^. Recently, neutrophil-to-lymphocyte ratio (NLR) has emerged as a potent composite inflammatory marker. Among the various inflammatory indicators, NLR is a sensitive inflammatory and prognostic indicator in many diseases including sepsis^14^, stroke^15^, cardiac disorders^16^, and cancer^17^ etc. Due to the similar inflammatory response in PQ poisoning, the NLR may also be used as prognostic indicator to predict mortality in patients with PQ poisoning. The aim of the present study is to investigate the prognostic value of the hematological parameters and neutrophil-lymphocyte ratio (NLR) in patients with acute PQ poisoning.

## Methods

### Ethics statement

This study complied with the guidelines of the Declaration of Helsinki and was approved by the Medical Ethics Committee of the First Affiliated Hospital, Anhui Medical University (Hefei, China). Since this study involved retrospective review of existing data, a waiver of written informed consent was obtained from the Institutional Review Board. All primary data was collected according to procedures outlined in epidemiology guidelines that strengthen the reporting of observational studies. Patient information was anonymized and de-identified prior to analysis.

### Patients

This retrospective cohort study included 202 patients with PQ poisoning was conducted in the emergency department (ED) from January 2010 to December 2015 at the First Affiliated Hospital, Anhui Medical University (Hefei, China). Diagnosis of PQ poisoning was based on clinical history, physical and laboratory examinations, especially urine sodium dithionite screening test. The qualitative urine PQ level was determined by the commercial test kit, which produced by the Syngenta (China) Investment Co., Ltd according to the urine dithionite test^18^. Briefly, ten milliliters of urine was placed into a beaker, 2 g of sodium bicarbonate was added, and the mixture was shaken gently. One gram of sodium dithionite was added and the mixture was shaken again. The mixture was viewed against a white background and compared with the standard colorimetric plate. The results were graded according to the color: barely distinguishable blue (+1), light blue (+2), deep blue (+3), and black (+4). We defined the positive urine PQ tests as the color grade ≥(+1). Patients were included in this study if they were older than 16 years of age and had urine PQ tests that showed positive. Patients were excluded from the study if the PQ exposure was limited to dermal exposure. They were also excluded if they did not have detectable PQ levels in their urine or if they had major comorbidities, such as chronic liver disease, chronic kidney disease, or diabetes. The diagnoses of major comorbidities were based on detailed clinical, physical, and laboratory examinations. All patients were managed under a standard clinical treatment protocol, which involves gastric lavage, catharsis, hemoperfusion, antioxidants, high-dose intravenous methylprednisolone and cyclophosphamide, and other treatment methods. As for hemoperfusion, patients have received 2 courses of 8 h active charcoal-containing hemoperfusion therapy with a 4 h interval within 24 h in the emergency intensive care unit (EICU). Hemoperfusion was administered through 2 femoral venous catheters at a blood flow rate of 200 mL/min. A single-use HA230 resin hemoperfusion apparatus (Zhuhai Jianfang Biotechnology Co., Ltd., Guangdong, China) was used which consisted of polypropylene housing material, cellulose, and activated charcoal adsorbent.

### Data collection

All data was collected using a standard collection form, which includes the following: (1) demographic parameters such as age and gender; (2) time interval from PQ ingestion to admit in the emergency department; (3) the estimated ingestion amount and plasma PQ level; (4) the initial vital signs including basal body temperature, heart rate, respiratory rate, and mean arterial blood pressure (MAP); (5) the laboratory parameters, including arterial pH, PaO_2_, PaCO_2_, leukocyte count, neutrophil count, lymphocyte count, platelet count, and levels of hemoglobin, albumin, bilirubin, alanine aminotransferase (ALT), blood urea nitrogen (BUN), creatinine, serum sodium, serum potassium within the first 24 h of admission; (6) Glasgow coma score (GCS), APACHE II, and SIPP score were recorded for each patient within 24 h after admission. The primary endpoint of this study was 30-day mortality. Patients were categorized into survivors or non-survivors group based on whether they survived after a 30-day follow-up.

### Statistical analysis

All of the statistical analyses were performed using SPSS 17.0 software (SPSS, Inc, Chicago, IL). Continuous variables were tested for normal distribution using the Kolmogorov-Smirnov test. Data were presented as mean ± standard deviation for normal distribution variables, or medians with range values for skewed distribution variables. The Student’s t test was used for normal distribution variables, whereas the Mann-Whitney U test was used for skewed distribution variables. Categorical variables were analyzed using the chi-square test. All risk factors were assessed by univariate Cox regression analysis, and variables that showed a *P* value less than 0.1 were included in a multivariate analysis by applying a multiple Cox regression. Receiver operating characteristic (ROC) curves were generated by plotting sensitivity against 1-specificity with the respective areas under the curves representing the predictive power of each parameter. The optimal cut-off value was represented highest Youden index (sensitivity + specificity − 1). Accuracy rate (AR) was calculated for each cut-off value. Survival analyses were estimated using the Kaplan–Meier method and were compared using the log-rank test. *P* value less than 0.05 was considered to be statistically significant.

## Results

### Patients’ characteristics

In present study, a total of 252 cases were screened from January 2010 to December 2015. Fifty cases were excluded, including 6 cases less than 16 years old of age, 10 transferred to other hospitals, 8 cases of patient discharge against medical advice, 5 cases of non-intended oral exposure, 15 cases of severe underlying diseases and 6 cases have miss data. Therefore, 202 patients were included in the analysis. The baseline characteristics of the patients are described in Table 1. In brief, the median age of patients was 32.00 (16.00–84.00) years, and 126 (62.38%) of the 202 patients were female. The median time interval between PQ ingestion and hospitalization was 6.50 (1.00–24.00) hours. The median ingested dose of 20% PQ was 20.00 (5.00–200.00) ml. The results of qualitative urine PQ level showed that 59 patients were graded as (+1), 89 patients were graded as (+2), 32 patients were graded as (+3), and 22 patients were graded as (+4) according to the urine dithionite test. One hundred and five patients (51.98%) died within 30 days due to acute PQ poisoning. The comparisons of parameters between non-survivors and survivors are shown in Table 2. The non-survivors were significantly older, hospitalized more quickly, and had a higher ingestion amount and plasma PQ level, mean arterial pressure (MAP), respiratory rate, creatinine, bilirubin, hemoglobin, albumin, but lower potassium levels upon admission. They also had lower partial pressures of carbon dioxide in arterial blood (PaCO_2_), and pH in their arterial blood at the same time point. The comparisons of complete blood count (CBC) between non-survivors and survivors also showed that non-survivors had significantly higher leukocyte, neutrophil counts, and NLR values, whereas there were lower lymphocyte counts and no significant difference in platelet counts. Moreover, the comparisons of traditional scores between non-survivors and survivors also showed that non-survivors had significantly higher SIPP and APACHE II score.

### Association of NLR and differential blood count with mortality

As shown in Table 3, univariate Cox regression analysis revealed that age, time to hospitalization, estimated ingestion amount, plasma PQ level, MAP, respiratory rate, creatinine, potassium levels, hemoglobin, albumin, pH, carbon dioxide in arterial blood (PaCO_2_), leukocyte, neutrophil, NLR, SIPP and APACHE II score were significantly associated with 30-day mortality. Simple linear regression indicated co-linearity between leukocyte counts and neutrophil counts. Therefore, leukocyte counts were not introduced into the multivariate Cox regression analyses. As shown in Table 4, multivariate Cox proportional hazards regression analyses revealed that estimated ingestion amount, time to hospitalization, NLR and APACHE II score were independent prognostic factors for 30-day mortality.

### Clinical predictors of mortality

**Figure 1:**
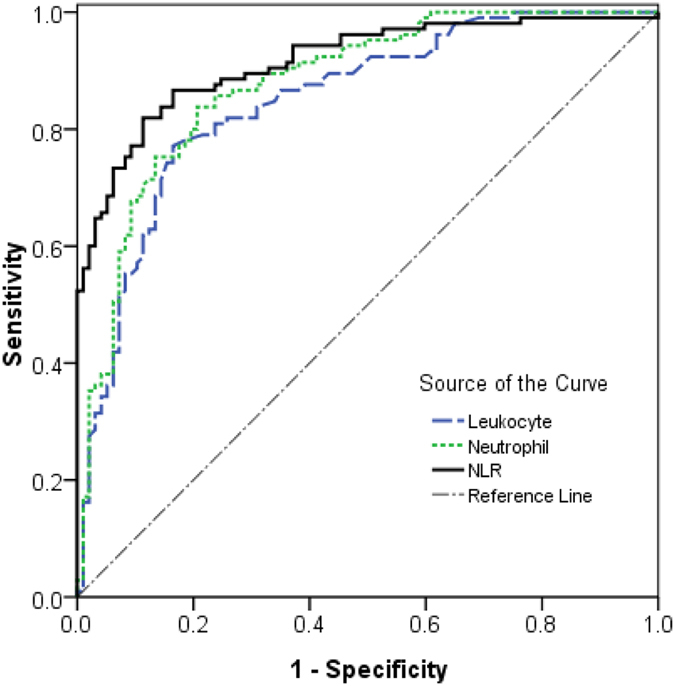
The ROC curves of leukocyte, neutrophil and NLR for predicting 30-day mortality.

**Figure 2:**
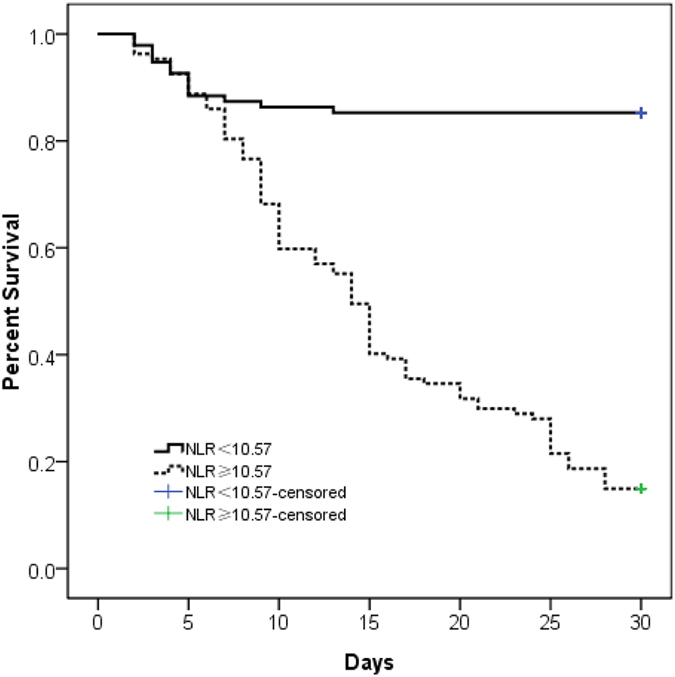
Kaplan-Meier survival curve stratified by NLR during diagnosis patients with acute PQ poisoning. *P* < 0.001 by the log-rank test.

For evaluating the prediction of NLR and differential blood count, we have used the ROC curve analysis. As shown in Table 5 and Fig. 1, the area under the ROC curve of the NLR value was 0.916(95%CI, 0.877–0.954) and the optimal cut-off value was 10.57 (sensitivity, 86.70%; specificity, 83.51%; Youden’s index, 0.702). The area under the ROC curve of the Neutrophil counts was 0.878(95%CI, 0.830–0.925) and the optimal cut-off value was 10.100 × 10^3^/mm^3^ (sensitivity, 83.80%; specificity, 79.38%; Youden’s index, 0.632). The area under the ROC curve of the leukocyte counts was 0.849(95%CI, 0.796–0.902) and the optimal cut-off value was 13.15 × 10^3^/mm^3^ (sensitivity, 77.10%; specificity, 83.50%; Youden’s index, 0.606). Moreover, a Kaplan-Meier curve was constructed to examine the relationship between NLR values and prognosis, the cumulative survival rates between patients with NLR < 10.57 (death rate 14.7%) and NLR ≥ 10.57 (death rate 85.0%) showed significant differences by using a log-rank test (Fig. 2).

For comparison of NLR and differential blood count with traditional prognostic predictors, we evaluated the prediction of plasma PQ levels, SIPP and APACHE II scores in 30-day mortality. As shown in Table 5, in the ROC curve analysis, the plasma PQ levels had an area of 0.885(95%CI, 0.839–0.931) and the optimal cut-off value was 2.48 μg/mL (sensitivity, 76.20%; specificity, 90.70%; Youden’s index, 0.669). The SIPP index had an area of 0.825(95%CI, 0.767–0.883) and the optimal cut-off value was 10.14 μg/mL/hour (sensitivity, 81.90%; specificity, 74.20%; Youden’s index, 0.561). The APACHE II score had an area of 0.876(95%CI, 0.828–0.923) and the optimal cut-off value was 7.50 (sensitivity, 69.50%; specificity, 90.72%; Youden’s index, 0.602). These results indicated plasma PQ levels, SIPP and APACHE II scores, also could be used to predict the prognosis of 30-day mortality in acute PQ poisoning.

## Discussion

Acute PQ poisoning is still a major public health problem in many developing countries, due to the high mortality and lack of effective treatment^19^. In present study, a total of 202 patients were included, the total mortality was 51.98% (105/202) during the 30-day follow-up. Generally, the severity and prognosis of acute poisoning are determined by the ingested dose. Several studies showed that plasma PQ concentrations are beneficial in predicting outcome for individuals with PQ poisoning^6^. However, serum PQ concentration detection is not available in most local hospitals, and the amount of ingestion is often difficult to estimate, particularly in patients presenting with confusion. For this reason, we sought to identify potential valuable and simple parameters for estimating prognosis of patients with acute PQ poisoning.

The complete blood count (CBC) is detected in almost all patients presenting with acute poisoning to the emergency department (ED). It is important to note that the test results are usually quick, inexpensive and routinely measured. Recently, there is study showed that the leukocyte and neutrophil counts had increased, whereas the lymphocyte counts had decreased, in several types of pesticide poisoning^20^. Moreover, Leukocyte, neutrophil counts, and NLR can estimate prognosis in patients with acute poisoning^20^. However, there is little knowledge of the application of CBC in the estimating prognosis of acute PQ poisoning. In present study, our results also clearly showed that the acute PQ poisoning patients had increased leukocyte and neutrophil, whereas had decreased lymphocyte counts. Furthermore, NLR, leukocyte counts, and neutrophil counts were strong prognostic indicator for predicting 30-day mortality. To the best of our knowledge, the present study is the first study that specifically explored the prognostic value of NLR, leukocyte counts, and neutrophil counts in PQ poisoning.

So far, the exact toxic mechanism of PQ poisoning remains unclear. However, several studies taking into account oxidative stress as the main mechanism of PQ-induced toxicity^21^. As a powerful redox cycling agent, intracellular PQ alternates the process of reduction and re-oxidation. Reactive oxygen species (ROS) are generated during this process and can cause subsequent cellular damage such as lipid peroxidation^22^. It has been reported that oxidative stress mediated disruption of CBC due to the free radicals induced chemokine production^23^. The peripheral leucocytosis is part of the more general response to stress, and neutrophil has been recognised as an early part of the inflammatory response^24^. Leukocytosis, neutrophilia, and lymphocytopenia can be detected in the acute clinical course when the oxidative stress is increased^25^. In present study, we also found that PQ could cause leukocytosis, neutrophilia, and lymphocytopenia in acute poisoning patients. Moreover, we found non-survivors had significantly higher leukocyte and neutrophil counts, whereas there was no significant difference in lymphocyte and platelet counts. Our results suggest that leukocytosis, neutrophilia, and lymphocytopenia may play a significant role in the toxicity of acute PQ poisoning.

Recently, a serial line studies have showed that the Neutrophil-lymphocyte ratio (NLR) is an indicator of prognosis in many diseases including acute poinsing^26^. NLR is a combination of these two biomarkers involved in the inflammatory process, which indicated the balance of the inflammatory and immune systems, making the NLR a useful index that reflects systemic inflammation responses. PQ has been previously reported to affect inflammatory and immune responses *in vitro*^27^. However, the prognostic value of NLR had not been investigated in the acute PQ poisoning patients. In the present study, we found that the non-survivors had significantly higher NLR. ROC curve analysis revealed that NLR was a strong prognostic indicator. By using a cut-off value 10.57 for NLR, sensitivity was 86.70% and specificity was 83.51% for prediction 30-day mortality. Moreover, our findings also suggested that the leukocyte and neutrophil counts had almost the same prognostic strength. ROC curve analysis for leukocyte and neutrophil counts revealed that these levels may be used for prediction 30-day mortality. By using a cut-off value 13.15 × 10^3^/mm^3^ for leukocyte counts, sensitivity was 77.10% and specificity was 83.50% for prediction 30-day mortality. By using a cut-off value 10.10 × 10^3^/mm^3^ for neutrophil counts, sensitivity was 83.80% and specificity was 79.38% for prediction 30-day mortality. Therefore, NLR, leukocyte and neutrophil counts may be useful and simpler parameters in assessing the prognosis of PQ poisoning.

As we known that some previous studies indicated plasma PQ levels and some traditional scores such as SIPP and APACHE II scores have good prognostic value for acute PQ poisoning^6^,^8^,^11^. In the present study, we also found plasma PQ levels, SIPP and APACHE II scores, were significantly higher in non-survivors than in survivors group. ROC curve analysis revealed that plasma PQ levels, SIPP and APACHE II scores, have prognostic value. By using a cut-off value 2.48 μg/mL for plasma PQ levels, sensitivity was 76.20% and specificity was 90.70% for prediction 30-day mortality. By using a cut-off value 10.14 μg/mL/hour for SIPP scores, sensitivity was 81.90% and specificity was 74.20% for prediction 30-day mortality. By using a cut-off value 7.50 for APACHE II score, sensitivity was 69.50% and specificity was 90.72% for prediction 30-day mortality. Therefore, plasma PQ levels, SIPP and APACHE II scores, also could be used to predict the prognosis of 30-day mortality in acute PQ poisoning, which consist with previous studies^6^,^8^,^11^. Moreover, the comparison of ROC analysis data of NLR with traditional prognostic markers showed that NLR may be have even better prediction efficiency than plasma PQ levels, SIPP and APACHE II scores at least in present study. Those results indicated NLR may be excellent prognostic predictor for acute PQ poisoning.

Some limitations and advantages of the present study should be addressed. Firstly, this study was conducted in a single institution, thus the results of the analysis may be subject to selective bias. However, our institution situates in middle region of China, which healthcare management also has been ranked in the middle level, therefore it could be used as a representative institution in China. Moreover, we have taken some methods to control selective bias: 1), we collected all patients’ information with acute PQ poisoning from January 2010 to December 2015 at our hospital, and selected with strict inclusion and exclusion criteria; 2) we have used cohort study to investigate the potential factors affecting 30-day mortality, which may ensure the survival and non-survival group patients come from the same population; 3) the sample size is relative large in the study of acute PQ poisoning, which also play important role in control selective bias; 4) last but not the least, we have retrospectively reviewed and collected the records of patients with acute PQ poisoning by two independent authors, which play important role in control of the other potential bias, such as information bias, in the present study. Secondly, we have compared the prediction of NLR and differential blood count with plasma PQ levels, and traditional scores such as SIPP and APACHE II scores, which have been indicated as good prognostic predictors for acute PQ poisoning. We found NLR, leukocyte and neutrophil counts may also be excellent prognostic predictors for acute PQ poisoning. Thirdly, several potential prognostic predictors recently have been indicated have prognostic value in acute PQ poisoning, including serum uric acid^28^, serum amylase activity^29^, plasma PTX3^30^, arterial lactate level and lactate metabolic clearance rate^7^, arterial blood gas analysis^31^, and so on. In contrast to those potential prognostic predictors, our present stduy have several advantages: 1) our results indicated NLR, leukocyte and neutrophil counts, may be have even better sensitivity and specificity by ROC analysis; 2) the complete blood count is a routine detection in hematological parameters, when patients presenting with acute poisoning to the emergency department; 3) it is important to note that the test results of complete blood count are usually quick, inexpensive and realized in almost all hospital, including local hospitals. Of course, hematological parameters, such as NLR, leukocyte and neutrophil counts, only reflect some aspects of the mechanism of acute PQ poisoning, therefore together hematological parameters with other potential prognostic biomarkers may be more reliable for evaluation the prognosis of acute PQ poisoning. Moreover, the present study was a retrospective investigation. Therefore, prospective investigations are still need to confirm those findings.

In summary, the present study shows that acute PQ poisoning can cause leukocytosis, neutrophilia, and lymphocytopenia. NLR, leukocyte and neutrophil counts have excellent prognostic value to the prediction 30-day mortality. Therefore, NLR, leukocyte and neutrophil counts may be valuable and simple parameters for evaluating prognosis in the patients with PQ poisoning.

## Additional Information

**How to cite this article**: Zhou, D.-C. *et al.* Prognostic value of hematological parameters in patients with paraquat poisoning. *Sci. Rep.* **6**, 36235; doi: 10.1038/srep36235 (2016).

**Publisher’s note:** Springer Nature remains neutral with regard to jurisdictional claims in published maps and institutional affiliations.

## Figures and Tables

**Table 1:** Baseline characteristics of PQ poisoning patients (n 003D 202).

Parameters	All patients
Age (years)	32.00(16.00, 84.00)
Gender(female)	126/202(62.38%)
Time to hospitalization (hours)	6.50(1.00, 24.00)
Estimated ingestion amount (mL)	20.00(5.00, 200.00)
MAP (mm Hg)	92.85 ± 13.69
Heart rate (beats/min)	80.50(47.00, 150.00)
Respiratory rate (breaths/min)	20.00(12.00, 45.00)
Body temperature	36.50(35.40, 40.00)
BUN (mg/dL)	5.49(1.84, 59.00)
Creatinine (mg/dL)	64.00(27.00, 182.00)
Sodium (mmol/L)	140.34 ± 5.21
Potassium (mmol/L)	3.50(2.00, 4.30)
ALT (U/L)	27.00(3.00, 109.00)
Bilirubin (mg/dL)	15.20(1.50, 80.00)
Hemoglobin (g/L)	138.00 ± 17.98
Albumin (g/L)	46.67 ± 6.71
pH	7.41(7.03, 7.74)
PaO_2_ (mm Hg)	87.48 ± 17.46
PaCO_2_ (mm Hg)	33.90(12.60, 58.10)
Leukocyte ( × 10^3^/mm^3^)	12.80(3.50, 45.40)
Neutrophil ( × 10^3^/mm^3^)	11.32(2.83, 40.77)
Lymphocyte ( × 10^3^/mm^3^)	1.10(0.10, 13.20)
Platelet ( × 10^3^/mm^3^)	197.08 ± 60.49
NLR	11.22(1.00, 77.94)
Plasma PQ level (μg/mL)	2.12(0.10, 58.50)
SIPP (μg/mL/hour)	12.97(0.33, 715.60)
APACHE II	6.00(0.00, 25.00)
30-day mortality	105/202(51.98%)

Data were presented as medians with range values for skewed distribution, means with SDs for normal distribution or number with percentage. Abbreviations: PQ, paraquat; MAP, mean arterial pressure; BUN, blood urea nitrogen; ALT, alanine transaminase; PaO_2_, partial pressure of oxygen; PaCO_2_, partial pressure of carbon dioxide; NLR, neutrophil-lymphocyte ratio; SIPP, the severity index of paraquat poisoning; APACHE II, the acute physiology and chronic health evaluation II score.

**Table 2:** The comparisons between survivors and non-survivors of PQ poisoning.

Parameters	Survivors (n = 97)	Non-survivors (n = 105)	*P* value
Age (years)	30.00(16.00, 74.00)	35.00(16.00, 84.00)	0.036
Gender(male/female)	33/64	43/62	0.310
Time to hospitalization (hours)	8.00(2.00, 24.00)	5.00(1.00, 22.00)	0.001
Estimated ingestion amount (mL)	17.00(5.00, 80.00)	50.00(5.00, 200.00)	<0.001
MAP (mm Hg)	89.03 ± 9.73	96.37 ± 15.77	<0.001
Heart rate (beats/min)	80.00(58.00, 129.00)	82.00(47.00, 150.00)	0.281
Respiratory rate (breaths/min)	20.00(16.00, 29.00)	20.00(12.00, 45.00)	0.043
Body temperature	36.50(35.80, 40.00)	36.50(35.40, 39.20)	0.136
BUN (mg/dL)	5.30(1.84, 42.80)	5.55(2.20, 59.00)	0.164
Creatinine (mg/dL)	57.00(32.00, 168.00)	75.50(27.00, 182.00)	<0.001
Sodium (mmol/L)	140.45 ± 4.05	140.24 ± 6.10	0.776
Potassium (mmol/L)	3.70(2.20, 4.30)	3.40(2.00, 4.20)	0.002
ALT (U/L)	27.00(6.00, 65.00)	28.00(3.00, 109.00)	0.919
Bilirubin (mg/dL)	14.59(1.50, 46.70)	16.75(4.40, 80.00)	0.041
Hemoglobin (g/L)	134.46 ± 17.84	141.27 ± 17.56	0.007
Albumin (g/L)	45.14 ± 6.54	48.10 ± 6.58	0.002
pH	7.42(7.33, 7.74)	7.39(7.03, 7.56)	<0.001
PaO_2_ (mm Hg)	87.06 ± 15.60	87.87 ± 19.08	0.742
PaCO_2_ (mm Hg)	35.70(14.40, 47.30)	31.60 (12.60, 58.10)	<0.001
Leukocyte ( × 10^3^/mm^3^)	9.50(3.50, 39.40)	17.90(7.50, 45.40)	<0.001
Neutrophil ( × 10^3^/mm^3^)	7.71(2.83, 33.45)	16.38(6.48, 40.77)	<0.001
Lymphocyte ( × 10^3^/mm^3^)	1.20(0.40, 9.80)	0.90(0.10, 13.20)	<0.001
Platelet ( × 10^3^/mm^3^)	198.71 ± 62.91	195.58 ± 58.43	0.714
NLR	7.16(1.00, 17.54)	17.89(1.51, 77.94)	<0.001
Plasma PQ level (μg/mL)	0.28(0.10, 16.47)	5.17(0.11, 58.50)	<0.001
SIPP (μg/mL/hour)	3.52(0.44, 260.40)	34.37(0.33, 715.60)	<0.001
APACHE II	3.00(0.00, 15.00)	10.00(0.00, 25.00)	<0.001

Data were presented as medians with range values for skewed distribution, means with SDs for normal distribution or number with percentage. Abbreviations: PQ, paraquat; MAP, mean arterial pressure; BUN, blood urea nitrogen; ALT, alanine transaminase; PaO_2_, partial pressure of oxygen; PaCO_2_, partial pressure of carbon dioxide; NLR, neutrophil-lymphocyte ratio; SIPP, the severity index of paraquat poisoning; APACHE II, the acute physiology and chronic health evaluation II score.

**Table 3:** Univariate Cox regression analysis of the risk factors for 30-day mortality.

Parameters	β Coefficient	Standard error	Hazard ratio	95% confidence interval	*P* value
Age (years)	0.023	0.006	1.023	1.011–1.035	<0.001
Gender(male/female)	−0.189	0.199	0.828	0.561–1.222	0.342
Time to hospitalization (hours)	−0.057	0.018	0.945	0.912–0.979	0.002
Estimated ingestion amount (mL)	0.043	0.003	1.044	1.037–1.051	<0.001
MAP (mm Hg)	0.030	0.007	1.030	1.017–1.044	<0.001
Heart rate (beats/min)	0.007	0.007	1.007	0.994–1.020	0.287
Respiratory rate (breaths/min)	0.095	0.029	1.099	1.039–1.163	0.001
Body temperature	−0.078	0.173	0.925	0.659–1.298	0.651
BUN (mg/dL)	0.017	0.012	1.017	0.994–1.041	0.146
Creatinine (mg/dL)	0.001	0.000	1.001	1.000–1.002	0.003
Sodium (mmol/L)	−0.007	0.020	0.993	0.955–1.033	0.722
Potassium (mmol/L)	−0.710	0.186	0.492	0.341–0.708	<0.001
ALT (U/L)	0.001	0.001	1.001	0.999–1.002	0.273
Bilirubin (mg/dL)	0.001	0.001	1.001	0.998–1.003	0.579
Hemoglobin (g/L)	0.015	0.006	1.015	1.004–1.026	0.007
Albumin (g/L)	0.051	0.017	1.053	1.019–1.087	0.002
pH	−6.763	1.095	0.001	0.000–0.010	<0.001
PaO_2_ (mm Hg)	0.004	0.006	1.004	0.992–1.016	0.524
PaCO_2_ (mm Hg)	−0.070	0.013	0.933	0.909–0.957	<0.001
Leukocyte ( × 10^3^/mm^3^)	0.084	0.010	1.088	1.068–1.109	<0.001
Neutrophil ( × 10^3^/mm^3^)	0.098	0.011	1.103	1.081–1.127	<0.001
Lymphocyte ( × 10^3^/mm^3^)	−0.080	0.116	0.923	0.735–1.159	0.491
Platelet ( × 10^3^/mm^3^)	0.000	0.002	1.000	0.997–1.003	0.823
NLR	0.040	0.005	1.041	1.030–1.052	<0.001
Plasma PQ level (μg/mL)	0.109	0.009	1.115	1.096–1.136	<0.001
SIPP (μg/mL/hour)	0.007	0.001	1.007	1.006–1.009	<0.001
APACHE II	0.204	0.017	1.226	1.186–1.268	<0.001

Abbreviations: PQ, paraquat; MAP, mean arterial pressure; BUN, blood urea nitrogen; ALT, alanine transaminase; PaO_2_, partial pressure of oxygen; PaCO_2_, partial pressure of carbon dioxide; NLR, neutrophil-lymphocyte ratio; SIPP, the severity index of paraquat poisoning; APACHE II, the acute physiology and chronic health evaluation II score.

**Table 4:** Multivariable Cox proportional hazards regression analysis of the risk factors for 30-day mortality.

Parameters	β Coefficient	Standard error	Hazard ratio	95% confidence interval	*P* value
Age (years)	0.008	0.008	0.992	0.978–1.007	0.313
Time to hospitalization (hours)	−0.127	0.030	0.881	0.831–0.934	<0.001
Estimated ingestion amount (mL)	0.036	0.006	1.037	1.025–1.048	<0.001
MAP (mm Hg)	0.010	0.009	0.990	0.973–1.007	0.253
Respiratory rate (breaths/min)	−0.048	0.036	0.953	0.889–1.022	0.175
Creatinine (mg/dL)	0.000	0.001	1.000	0.998–1.002	0.869
Hemoglobin (g/L)	0.006	0.007	1.006	0.992–1.021	0.388
Albumin (g/L)	0.009	0.021	1.009	0.968–1.052	0.660
pH	−0.335	1.235	0.715	0.064–8.054	0.786
PaCO_2_ (mm Hg)	0.009	0.014	1.010	0.982–1.038	0.497
Neutrophil( × 10^3^/mm^3^)	0.008	0.019	1.008	0.971–1.047	0.665
NLR	0.033	0.008	1.033	1.017–1.049	<0.001
Potassium (mmol/L)	−0.052	0.146	0.949	0.713–1.264	0.721
Plasma PQ level (μg/mL)	0.000	0.019	0.999	0.963–1.037	0.975
SIPP (μg/mL/hour)	0.003	0.002	1.003	0.998–1.007	0.221
APACHE II	0.187	0.039	1.205	1.115–1.302	<0.001

Abbreviations: PQ, paraquat; MAP, mean arterial pressure; PaCO_2_, partial pressure of carbon dioxide; NLR, neutrophil-lymphocyte ratio; SIPP, the severity index of paraquat poisoning; APACHE II, the acute physiology and chronic health evaluation II score.

**Table 5:** Prediction of the mortality by ROC curve in acute PQ poisoning.

Predictive factors	AUC (95% CI)	*p* Value	Cutoff point	Sensitivity (%)	Specificity (%)	Youden index	Acuracy rate (%)
NLR	0.916 (0.877–0.954)	<0.001	10.57	86.70	83.51	0.702	85.15
Neutrophil ( × 10^3^/mm^3^)	0.878 (0.830–0.925)	<0.001	10.10	83.80	79.38	0.632	81.68
Leukocyte ( × 10^3^/mm^3^)	0.849 (0.796–0.902)	<0.001	13.15	77.10	83.50	0.606	80.39
Plasma PQ level (μg/mL)	0.885 (0.839–0.931)	<0.001	2.48	76.20	90.70	0.669	83.45
SIPP (μg/mL/hour)	0.825 (0.767–0.883)	<0.001	10.14	81.90	74.20	0.561	78.05
APACHE II	0.876 (0.828–0.923)	<0.001	7.50	69.50	90.72	0.602	80.12

Abbreviations: PQ, paraquat; AUC, area under receiver-operating characteristic curve; CI, confidence interval; NLR, neutrophil-lymphocyte ratio; SIPP, the severity index of paraquat poisoning; APACHE II, the acute physiology and chronic health evaluation II score.
